# Botulinum Toxin Type A for Trigeminal Neuralgia: A Comprehensive Literature Review

**DOI:** 10.3390/toxins16110500

**Published:** 2024-11-20

**Authors:** Yan Tereshko, Simone Dal Bello, Christian Lettieri, Enrico Belgrado, Gian Luigi Gigli, Giovanni Merlino, Mariarosaria Valente

**Affiliations:** 1 Clinical Neurology Unit, Udine University Hospital, Piazzale Santa Maria della Misericordia 15, 33100 Udine, Italy; yan.tereshko@asufc.sanita.fvg.it (Y.T.); simonedalbello@libero.it (S.D.B.); christian.lettieri@asufc.sanita.fvg.it (C.L.); giovanni.merlino@asufc.sanita.fvg.it (G.M.); mariarosaria.valente@uniud.it (M.V.); 2 Neurology Unit, Udine University Hospital, Piazzale Santa Maria Della Misericordia 15, 33100 Udine, Italy; enrico.belgrado@asufc.sanita.fvg.it; 3 Department of Medicine (DAME), University of Udine, Via Colugna 50, 33100 Udine, Italy

**Keywords:** botulinum toxin type A, trigeminal neuralgia, review

## Abstract

Trigeminal neuralgia is a neuropathic pain syndrome responsive to botulinum toxin type A therapy. This review had the goal of analyzing the different studies published from 2002 to January 2024 to better define the techniques and the types of botulinum toxin type A used, the doses, the injection routes, and the different populations of trigeminal neuralgia patients treated. We considered only articles in which the therapy was administered to humans to treat trigeminal neuralgia. Case reports, case series, open-label, retrospective, and RCT studies were considered. The research was conducted on MEDLINE and the keywords included (trigeminal neuralgia) and (botulinum). Thirty-five articles were considered suitable for this review. Botulinum toxin type A was shown to be an effective therapy for TN pain in all the articles analyzed, albeit there is a lack of standardization in methods and outcomes. The techniques, the doses, and the injection approaches were very heterogeneous among the studies. Only two botulinum toxin type A formulations have been used in this setting: onabotulinumtoxinA and lanbotulinumtoxinA. There were 300 patients treated with onabotulinumtoxinA and 760 treated with lanbotulinumtoxinA overall (in 42 patients, the formulation was not specified). The distinction between etiological and clinical types of TN has been made by only a small portion of the studies. The main adverse event was transient facial asymmetry. Botulinum toxin type A is indeed a promising therapy that is clearly effective for trigeminal neuralgia. OnabotulinumtoxinA is the most common formulation used in Western countries; however, the meager sample of TN patients treated, and the lack of standardization are not sufficient for this therapy to be approved by the FDA or EMA. Indeed, more studies with standardized methods and larger samples are needed for this purpose.

## 1. Introduction

Trigeminal neuralgia (TN), or tic douloureux, is a chronic pain syndrome characterized by recurrent paroxysmal episodes of brief, severe, unilateral pain in one or more trigeminal nerve branches. The lifetime prevalence of TN is 0.16–0.30%, and there are 4–13 cases per 100,000 people each year. Most cases have an onset after the age of 50, and more females are affected than men [1,2]. Each paroxysm has abrupt onset and termination and lasts from a few seconds to 2 min in 74% of cases. The frequency may range from one to over 50 attacks a day, with 40% of TN patients experiencing more than ten paroxysmal attacks per day [3,4]. The pain is mainly described as stabbing, shock-like, lancinating, or burning. Only one nerve branch is involved in 60% of cases, while in 35%, two branches are involved. The ophthalmic branch is involved in only 4% or less of patients with trigeminal neuralgia. In these cases, mild autonomic symptoms can be associated, such as rhinorrhea, lacrimation, hypersalivation, facial flushing, and conjunctival injection [5]. If these associated autonomic symptoms are intense and frequent, and only the V1 branch is involved, the diagnosis of short-lasting unilateral neuralgiform headache attacks (SUNHA) must be considered. Although most patients have only one branch involved, a progressive involvement of other branches can be observed.

Moreover, the affected division, the side of the face, and the pain quality may change throughout the disease course [6,7,8]. When TN involves two nerve branches, they must be contiguous. The physician must ascertain that pain does not extend to the back of the ear, the angle of the mandible, and the posterior third of the skull, since extra-trigeminal cranial nerves innervate these areas. Bilateral involvement is rare and is often related to secondary causes, such as multiple sclerosis or growing tumors of the cerebellopontine angle [9,10]. Pain can be spontaneous or provoked by the stimulation of trigger points located in the nerve branch or branches involved. The most common triggers are the activation of masticatory muscles, tooth brushing, cold air, and touching of facial skin. Light tactile stimulation is the most potent trigger, while painful and thermal stimulation are usually ineffective [11]. These trigger points are frequently located in the nasal or perioral regions near the midline of the face, and include the nasolabial fold, the upper lip, the lateral aspect of the lower lip, the chin, the cheek, and the alveolar gingiva [12,13,14].

Moreover, patients with exclusively spontaneous pain are virtually unknown, and evoked pain is reported in 99% of patients [14,15,16], showing a high diagnostic value in this setting [17]. Pain episodes are followed by a refractory period that lasts a variable amount of time [18], and this varies among trigeminal autonomic cephalalgias such as SUNHA. When pain-free periods become very brief and pain episodes become very frequent, quality of life and mood are severely impaired. Interestingly, painful attacks usually do not present during sleep. An interesting feature of TN, different from other forms of neuropathic pain, is that it enters into a period of complete remission in up to 63% of patients. This period could last weeks or years [14,19]. Sensory deficits may be found during neurological examination in 30% of cases; however, in most cases, they are subtle and detectable only with quantitative sensory testing [14,17,20]. Although the absence of sensory deficits does not rule out secondary TN, sensory discrimination deficits are highly indicative of secondary TN [21]. In classical and secondary TN, demyelination and possibly secondary axonal loss are the main mechanisms involved in the pathophysiology of TN pain [22,23,24,25,26,27,28]. Demyelination could lower excitability thresholds and promote ephaptic propagation towards nearby fibers because the myelin sheath is damaged [29,30,31,32]. There is evidence of abnormal expression of NaV1.7, NaV1.3, and NaV1.8 sodium channels [33], as well as potassium voltage-gated channels [34]. This leads to the dysregulation of resting potentials, facilitating the spontaneous and stimulated repetitive firing of action potentials. The most used and effective medical treatment options are carbamazepine and oxcarbazepine, which provide pain relief in almost 90% of patients. However, in about 40% of cases, there are significant adverse events causing therapy withdrawal [35]. There is less evidence and less efficacy for other therapeutic options, including baclofen, lamotrigine, gabapentin, and pregabalin, than for sodium channel blockers. Moreover, these drugs can cause serious adverse events. Radiofrequency ablation, chemodenervation, balloon compression, and radiosurgery are interventional therapies that might lead to unpleasant or severe adverse events and can sometimes not achieve adequate pain relief [36,37,38,39,40,41,42,43,44,45].

The therapeutic use of botulinum toxin was established in 1970 to treat strabismus [46]. Its use extended to the treatment of dystonia, hemifacial spasms, neurogenic detrusor activity, primary hyperhidrosis, and to cosmetic purposes [47]. In 1986, its effect on pain was reported in a group of patients affected by cervical dystonia [48]. Since then, numerous papers and case reports have investigated its anti-nociceptive effect. To date, chronic migraine is the only approved pain indication of botulinum toxin type A (BoNT/A). Still, the evidence of its efficacy in other pain syndromes, such as trigeminal neuralgia, is well-established [49,50]. Moreover, BoNT/A has an excellent profile regarding safety and tolerability and is generally devoid of serious adverse events [51]. Botulinum toxin type A might be an excellent therapeutical option for TN patients who cannot use conventional oral drugs due to adverse events or scarce tolerability and are not eligible or eager to undergo interventional procedures.

### 1.1. Botulinum Toxin Type A

Botulinum neurotoxin (BoNT) is produced by a gram-positive anaerobic bacillus known as Clostridium botulinum and is among the most lethal biological toxins in nature. This toxin has seven serotypes classified with a capital letter from A to G. Each serotype has its own unique molecular structure and mechanism of action, but only serotypes A, B, E, and F can cause botulism in humans. Type A and Type B serotypes are used in clinical practice. BoNT has a molecular weight of 150 KD and is composed of a light chain (LC) of 50 KD and a heavy chain (HC) of 100 KD, linked by a disulfide bond. LC presents a catalytic domain, while HC has a binding and translocation domain [52]. BoNT is associated with a protein complex (non-toxic neurotoxin-associated proteins or NAPs) to form a neurotoxin complex. NAPs include hemagglutinin proteins (HAs), which are involved in the translocation of the complex from the intestinal epithelial lining into the bloodstream and lymphatic system, and non-hemagglutinin proteins (NAHs), which provide stability and protection to the complex against protease in an acid environment [53]. Autoantibodies can be formed against HA (in particular against NAP-33). In muscle with a neutral pH, BoNT and NAPs dissociate and the neurotoxin is activated by itself (Type A) or by a tissue protease (other serotypes). Within several hours, botulinum toxin enters the peripheral nerve terminals. The HC binds to a specific pre-synaptic membrane receptor; for example, serotype A binds to SV2A-C, while serotype B binds to synaptotagmins I and II. This bond induces BoNT internalization in an endosome where the HC and LC separate and HC opens a channel in the endosome through which the LC translocates in the cytosol. The LC cleaves the proteins of the SNARE complex (formed by the heterotrimers VAM2, SNAP25, and syntaxin) and prevents pre-synaptic vesicle fusion and the release of neurotransmitters such as acetylcholine. Each serotype has a specific target on the SNARE complex: serotype A cleaves SNAP25, while serotype B catalyzes synaptobrevin (VAMP2). BoNT serotype A (BoNT/A) protease is concentrated along the inner surface of the plasma membrane in proximity to septins; moreover, the toxin seems to recruit enzymes that remove polyubiquitin chains. This permits BoNT/A to survive for up to 5 months in neurons, while the neuromuscular junction functionality recovers in 3–4 months. BoNT/A cleaves SNAP25 and forms a truncated product named SNAP25 (1-197). This molecule does not inhibit SNARE complex formation (VAM2, SNAP25, and syntaxin), and the recombinant complex competes with normal SNARE complexes. A small percentage of cleaved SNAP25 (2–20%) can block the entire synapsis.

The diffusion of the toxin is dose-dependent. In an experiment of 1994, 1 onaA U was injected into the longissimus dorsi muscle of a rabbit, and the spread of it was within 15–30 mm; when 5–10 onaA U were injected, the toxin spread into the entire muscle.

### 1.2. Botulinum Toxin Type A Formulations

Four BoNT formulations are available in clinical practice in the USA and Europe. Botulinum serotype A toxins are onabotulinumtoxinA (Botox^®^—Allergan, Inc., Irvine, CA, USA; 100–200 U vials), incobotulinumtoxinA (Xeomin^®^—Merz Pharmaceuticals LLC, Greensboro, NC, USA, 50–100 U vials), and abobotulinumtoxinA (Dysport^®^—Ipsen Biopharm Ltd., Wrexham, UK; 300–500 U vials). These three botulinum toxins need to be prepared and diluted with 0.9% sodium chloride. Rimabotulinum toxin B (Myobloc in the USA and NeuroBloc in Europe—Solstice Neurosciences, Inc., San Francisco, CA, USA; 2500–5000–10,000 U vials) is the only serotype B BoNT used in clinical practice and comes already prepared in a diluted vial. The unit equivalence of these different botulinum toxins is as follows: 1 U onaA = 1 U incoA = 3–4 U aboA = 40–50 U rimaB. All the BoNT vials need to be stocked in a refrigerator, except for incoA. All these toxins must be used within 24 h of their preparation. Moreover, the three A serotypes have similar diffusion patterns. LanbotulinumtoxinA was introduced in China and then worldwide under different brand names (Hengli^®^, Prosigne^®^, Lantox^®^, Lazox^®^, Redux^®^, Liftox^®^, BTX-A, and HTBX-A). Its components are similar to onabotulinumtoxinA and abobotulinumtoxinA, but the stabilizing protein used is made of bovine gelatin (5 mg per vial) and not of human serum albumin. Regarding the potency of this lanbotulinumtoxinA, a recent study compared it to onabotulinumtoxinA and incobotulinumtoxinA and did not report significant differences; therefore, a 1 U lanA = 1 onaA ratio is suggested [54].

### 1.3. Mechanisms of Action of Botulinum Toxin Type A on Pain

BoNT/A seems to have selective entrance into capsaicin-sensitive sensory neurons; the TRPV1-expressing neurons in both the peripheral and central nervous systems have a high affinity to BoNT/A [55]. Therefore, only pain-related mechanical and inflammatory sensation are affected. It is also possible that sensitized-only neurons express a high concentration of SV2A, which might facilitate the entrance of the toxin [56]. When injected into the spinal canal, the effect of BoNT/A on pain starts earlier (24 h) and requires lower doses when compared to subcutaneous injections (3–4 days). BoNT/A can also inhibit the release of other neurotransmitters, such as CGRP, substance P, ATP, glutamate, serotonin, and noradrenaline in both the central and peripheral nervous systems [57,58,59]. This is probably due to their dependence on the SNARE complex for release.

Moreover, BoNT/A has a central anti-nociceptive effect, perhaps due to retrograde axonal and trans-synaptic transport [60,61,62]. The injection of BoNT/A into rats’ whisker pads showed the presence of cleaved SNAP25 in the pars oralis and caudalis of the trigeminal nucleus. Some authors have described an indirect effect of BoNT/A on distant sensory regions in orofacial pain induced by formalin. For example, c-Fos expression in the PAG and locus coeruleus has been shown to be diminished, suggesting a pain modulatory effect on the ascending sensory areas. The expression of TRPV1 and P2X3, but not their mRNA, was found to be significantly reduced in the sensory ganglia of damaged nerves with BoNT/A injection; this suggests that botulinum toxin might block TRPV1 translocation on the neuronal membrane surface of the sensory ganglia [63,64,65]. Moreover, the axonal transport blocker colchicine blocks the effect on pain of BoNT/A; the toxin inhibits the exocytosis of vesicles containing TRPV1. The reduced expression of TRPV1 following BoNT/A injections has been demonstrated in sensory neurons projecting from the dura mater, corroborating the theory of axonal retrograde transport and trans-synaptic translocation between different sensory neurons [60,62,66,67]. The antinociceptive effect of BoNT/A, when injected into one side, also affects the contralateral side in many bilateral pain models [68,69].

Interestingly, BoNT/A could restore normal opioidergic transmission in the trigeminal nucleus’s pars caudalis and the spinal cord’s dorsal horn. For example, in sciatic pain models of rats, BoNT/A enhanced GABA-A and μ-opioid receptor transmission, increasing the analgesic response and tolerance to morphine [70,71,72,73]. BoNT/A affects the regeneration of injured peripheral nerves and interferes with neuroinflammation. Neuropathic mice were injected with BoNT/A in the paw, leading to an increase in cdc2 expression and Schwann cell proliferation [64,74]. Furthermore, the toxin may be transported in a retrograde fashion through axons and into Schwann cells [61].

Interestingly, BoNT/B does not have this ability [75]. Intraplantar injections of BoNT/A in rats reduced ipsilateral microglia activation and astrocyte activation in the dorsal and ventral horns of the spinal cord [64,76]. In another study on rats, BoNT/A injections reduced microglia activation by IL-1β, and IL-18, and enhanced the expression of IL-1RA and IL-10 in the spinal cord and DRG [77]. In vitro, BoNT/A affected the functioning of p38, ERK1/1, and NF-κB, and reduced the expression of IL-1β, IL-18, IL-6, and NOS2; it also diminished SNAP23 activity and enhanced TLR2 expression [78].

BoNT/A’s peripheral anti-inflammatory effects are debated, due to contradictory results from various experiments [79]. In models of cystitis and prostatitis induced by cyclophosphamide and capsaicin, respectively, BoNT/A was able to decrease neurogenic inflammation and hypersensitivity in the bladder and the accumulation of inflammatory cells and COX2 expression in the prostate [80,81]. Moreover, the articular injection of BoNT/A in models of acute and chronic arthritis reduced IL-1 β and TNF- α in the synovial tissue, bringing about an improvement in cartilage degeneration and inflammation [82,83]. On the other hand, in acute inflammatory pain induced by intraplantar injection of capsaicin or carrageenan, BoNT/A preventive injections could not affect carrageenan-induced edemas or capsaicin-induced protein extravasation from the bloodstream [84].

## 2. Results

There are 35 articles on the efficacy of botulinum toxin type A in humans with TN. Ten studies are case reports or case series, 17 are open-label studies (one randomized), four are RCTs, and four are retrospective studies. The list of the studies and their findings is shown in Table 1.

## 3. Discussion

### 3.1. Botulinum Toxin Type A Formulation, Dilution, Dose, and Injection Technique

OnabotulinumtoxinA was used in 18 studies (including five case reports or case series), while LanbotulinumtoxinA was used in 11 studies (including one case report). The remaining six studies (including four case reports and two open-label studies) did not report the formulation name of the botulinum toxin type A. AbobotulinumtoxinA or incobotulinumtoxinA were not used in the setting of trigeminal neuralgia in any research paper examined. In twelve articles, the authors did not report the dilution of BoNT/A. OnabotulinumtoxinA was reconstituted with sodium chloride 0.9% to obtain a dilution of 50 U/mL in most cases (eight studies), and only one study reported a dilution of 100 U per ml; in one case series and one case report, the authors reported a dilution of 25 U/mL and 40 U/mL, respectively. LanbotulinumtoxinA was reconstituted with sodium chloride at 0.9% to obtain a dilution of 50 U/mL in six studies; in two studies, the dilution was 25 U/mL, and in the other two studies, it was 100 U/mL. In the studies in which the BoNT/A manufacturer was not specified, the dilution was reported in only two case reports (50 U/mL and 100 U/mL). Moreover, the dose and the injection techniques used in the literature are heterogeneous: the doses range from 5 U to 200 U. The injections were mostly performed with an intradermal and/or subcutaneous approach (10 studies) or an intradermal/subcutaneous and intraoral approach (11 studies); in one article, the authors used the intraoral approach in only one study. In four studies, the authors adopted an intramuscular-only injection technique, while in two other studies, the authors combined intramuscular with subcutaneous injections or intraoral approaches. In one study, the authors injected BoNT/A with a near-nerve technique, while in two other studies, the botulinum toxin was injected into the sphenopalatine ganglia. In four studies, the approach was not reported at all.

**Figure 1:**
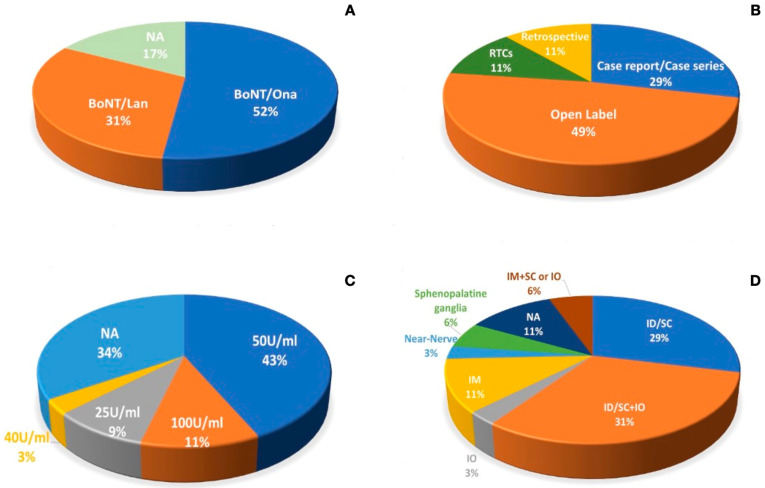
(**A**) shows the percentage of the type of botulinum toxin used in the literature, while (**B**) shows the research design of the articles examined in this review. (**C**) describes the dilution, and (**D**) represents the distribution of injection approaches used in the articles examined. Legend: NA—not available; IM—intramuscular; SC—subcutaneous; ID—intradermal; IO—intraoral.

It is important to specify that in one open-label study, the authors injected 50 U of onabotulinumtoxinA in two sites, one above and one below the zygomatic arch, without the specification of whether these injections were intramuscular or intradermal/subcutaneous. The “follow the pain” principle was used in most cases to treat the patients; in a minority of studies, the treatment was performed following the course of the trigeminal nerve branches, and the trigger zones were treated in most cases. See Table 1 for the details of the studies and Figure 1 for the data distribution.

### 3.2. Botulinum Toxin Type A Efficacy on Humans

Despite the overall efficacy of BoNT/A reported in case reports/case series and research papers, only a few papers reported the efficacy of the treatment, referring to a reduction in pain of at least 50%. Overall, the examined studies are heterogeneous, with different BoNT/A formulations, doses, treatment protocols, and follow-ups; the lack of standardization makes it difficult to quantify the efficacy of BoNT/A correctly.

Four RCT placebo-controlled studies reported significant improvement in the TN patients treated with BoNT/A compared to the placebo group [94,95,96,99]. In two of these RCTs, the experimenters used lanbotulinumtoxinA with intraoral or intradermal injections, while the remaining two used onabotulinumtoxinA with subcutaneous injections; moreover, these studies used different doses, dilutions, and sites of injection. They treated idiopathic, classical, or idiopathic and classical TN patients; no secondary forms were treated, and no distinction was made between type 1 and type 2 TN. The sample size was also heterogeneous, ranging from 20 to 84 patients; most patients were treated with lanbotulinumtoxinA (77 out of 124), while only a few were treated with onabotulinumtoxinA (30 out of 56). These data make these studies difficult to compare; first of all, due to the different formulations of botulinum toxin type A used. Despite these considerations, in all of these studies, botulinum toxin type A was superior to placebo. The most common side effect observed was facial asymmetry, occurring in 12 of the 107 treated patients (11.2%).

The treatment efficacy (>50% pain reduction) was 92% at 1-month follow-up, 100% at 2-month follow-up, and 77% at 3-month follow-up in one study; moreover, in the same study, the treatment was effective in 38.6% of patients at 14-month follow-up [97]. However, this extended period of pain relief is possibly due to the typical spontaneous remission of TN and not due to the botulinum toxin effect. Overall, most papers reported the mean and SD of pain and frequency data at different follow-up phases. In six research papers, the improvement was not quantified (three case reports or case series and three open-label studies). Nonetheless, the authors reported significant pain reduction. Moreover, in three case reports, pain was resolved completely. The duration of the antalgic effect after the first round of injections in case reports/case series ranges between 60 days and 5 months; in open-label studies and RCTs, the duration ranges between 2–3 months and 14 months. Interestingly, the studies with lanbotulinumtoxinA reported a longer duration of the antalgic effect than the onabotulinumtoxinA studies (3–6 months–14 months vs. 2–3 months–6 months); however, these data could be related to a remission period of TN, since the effect of BoNT/A on pain lasts typically 3–6 months.

In most studies, the therapeutic effect began 1–2 weeks post-injection; this delay between injection and pain relief likely reflects the time needed for the toxin molecule to enter the presynaptic nerve terminal and travel along the axon. However, not all BoNT/A molecules may be transported centripetally; there appear to be differences in the intracellular processing of BoNT/A molecules related to their size. Specifically, the larger BoNT/A fraction seems to enter acidic vesicles, leading to rapid translocation into the cytosol. In comparison, the smaller fraction appears to enter non-acidic vesicular compartments and is then transported centripetally via microtubule-dependent retrograde axonal pathways [120].

Considering all the studies we examined, 300 patients were treated with onabotulinumtoxinA, 760 patients were treated with lanbotulinumtoxinA, while in 42 patients, the BoNT/A formulation was not specified.

### 3.3. Correlation Between Dose and Pain Reduction

In a retrospective study with 152 TN patients treated with BoNT/A, there was a non-significant correlation between the dose and the degree of pain relief [112]. In other studies, there were no correlations between the dose and the degree of pain reduction [94,97,99,107]; however, one open-label study reported a positive correlation between the dose and the earlier onset of action [91]. The higher doses could be related to the longer duration of BoNT/A [112].

### 3.4. Etiologic and Clinical TN Differences

The analyzed studies paid limited attention to distinguishing the different clinical and etiological characteristics of trigeminal neuralgia treated with type A botulinum toxin. Only 14 of the 35 studies reviewed reported the clinical type (type 1 or type 2) and etiology (idiopathic, classical, or secondary) of the trigeminal neuralgia treated. A total of 18 articles provided partial information, indicating only the etiology but not the clinical type, while three articles reported no details on either the clinical type or etiology. This lack of data is significant, given the potential variations in treatment response according to different etiologies and clinical types. Future studies must address patient characteristics more rigorously to identify possible differences in response to botulinum toxin or other available treatments.

### 3.5. Adverse Events

Facial asymmetry is the most common adverse event in this setting and was reported in 20 research papers out of 35; in five studies, the authors noted the absence of facial asymmetry, while in seven studies, adverse events were not reported. Other adverse events include dysesthesia, hematoma, itching, edema, and local swelling; their incidences were very low. In the two research papers involving the treatment of the sphenopalatine ganglia with BoNT/A, adverse events were reported in only one of them, including swelling, jaw dysfunction, dry eye, diplopia, cutaneous rash, and dysphagia. In two studies, adverse events were reported but not specified.

## 4. Conclusions

Botulinum toxin type A is an effective therapy for trigeminal neuralgia. However, only 300 TN patients were treated with onabotulinumtoxinA, and 760 patients were treated with lanbotulinumtoxinA. In 42 patients, the formulation of botulinum toxin type A was not specified. In Western countries, clinicians use onabotulinumtoxinA and not lanbotulinumtoxinA; this implies that there is a need for more studies, RCTs, and a larger number of TN patients treated for the approval of the drug in clinical settings. In all the studies analyzed, BoNT/A effectively treated the pain of TN patients. However, the data regarding etiology and clinical type differences are insufficient; many studies did not report whether the TN was classical, idiopathic, or secondary.

Nevertheless, the clinical type (type 1 or 2) was frequently unreported. These data might be necessary since the etiological and clinical types may respond differently; very few studies compared the different clinical and etiological types of TN, and the results were discordant. There was also significant heterogeneity in the injection routes and doses used in various studies, and a clear comparison is complex; in some cases, the researchers performed near-nerve or sphenopalatine ganglia injections, which are difficult to perform in a clinical setting or performed injections without specifying where the BoNT/A was injected.

The subcutaneous/intradermal technique was the most used, easiest, and fastest method on clinical and research grounds. Intraoral and intramuscular injections were also performed; however, it is unclear whether one injection method is more effective and safer than others. The doses injected in each patient were very heterogeneous, ranging from very few units to hundreds. Interestingly, the outcome was mainly focused on pain intensity reduction on NRS or VAS grounds, and only a few articles recorded the frequency of paroxysms. Finally, the most common adverse event of BoNT/A therapy is facial asymmetry, which is always transitory and is also mild in most cases. The lack of standardization and the heterogeneity of the methods make it difficult to compare the results between the different studies and perform adequate meta-analysis. Moreover, in some studies, the lack of differentiation between type 1 and type 2 TN or between the different TN etiologies, does not permit us to understand whether one group could respond better than another.

Indeed, Botulinum toxin type A is a promising therapy that is effective in treating trigeminal pain. In this regard, future studies must include the TN etiology (classical, idiopathic, or secondary) and clinical type (1 or 2), and describe the BoNT/A formulation, dilution, dose, and injection technique. The outcome must take into consideration not only pain intensity reduction (with the NRS or VAS scales) of painful paroxysms but also their frequency and the baseline pain intensity of type 2 TN patients. A comparison between TN etiological and clinical types must be performed. We prefer subcutaneous/intradermic BoNT/A (onabotulinumtoxinA) injections since they are fast and easy to perform in the outpatient clinic setting, and the subcutaneous layer is rich in A-delta and C fibers; using a dilution of 100 U/mL might reduce the spread of the toxin into the lower muscular layers of the face, leading to lower rates of facial asymmetry. To standardize the injection sites, we recommend performing the injections virtually following the route beneath the skin of the trigeminal branches, with 4–5 injections per branch and 5 U of BoNT/A per site; an extra 5 U might be injected in the trigger zones.

More studies with standardized methods and larger samples are needed to approve its use in this setting.

## 5. Methods

The online literature search was conducted using MEDLINE (Pubmed) to find case reports, case series, open-label, retrospective, and RCT studies. We considered only the original articles published in indexed journals in which botulinum toxin was used on humans to treat trigeminal neuralgia; we analyzed the botulinum formulation and type, the dose, the route of administration, and the sites treated; we also evaluated whether the patients presented with type 1 or type 2 neuralgia and if they had idiopathic, classical, or secondary forms. We analyzed the modality of evaluation of the efficacy of the treatment, and we collected and correlated the adverse events. We excluded studies on animals, abstracts, reviews, guidelines, studies in which data from trigeminal neuralgia patients were pooled indistinctively together with those of other neuropathic patients, study protocols, commentaries, and correspondences.

Keywords used for the search included the following: (trigeminal neuralgia) and (botulinum).

The search was not limited to the English language; we included articles in Chinese, Russian, and Spanish only languages. The articles analyzed were published up till January 2024.

**Figure 2:**
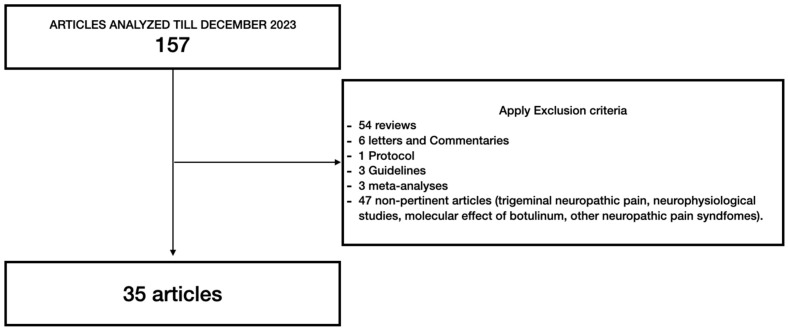
Comprehensive literature review flowchart.

A total of 157 results were found. Fifty-four articles were reviews, forty-seven articles were considered not pertinent (trigeminal neuropathic pain, other neuropathic pain syndromes, molecular evaluations, and neurophysiological studies), eight articles involved animals, six articles were letters or commentaries, one article was a protocol, three articles were guidelines, and three articles were meta-analyses. Thirty-five articles were considered suitable for this review (see Figure 2).

## Figures and Tables

**Table 1:** The list of studies, in chronological order, regarding the efficacy of BoNT/A on humans with TN. Legend: N/A—not available; IM—intramuscular; VAS—visual analogic scale; SC—subcutaneous; TN—trigeminal neuralgia.

	Study Type	Toxin Type and Dilution, Injection Route, and Dose	Patients	Major Findings	Adverse Events
[85] Micheli et al., 2002	Case report	Type: onabotulinumtoxinA Dilution: N/A Approach: IM Dose: 2.5 per site (two sites) Location: orbicular oculi, buccinator muscles	One patient with painful convulsive tic (hemifacial spasm and Type 1 classical TN)	Pain ameliorated for 10 weeks and then reappeared. The procedure was repeated every 12 weeks with significant improvement.	N/A
[86] Allam et al., 2005	Case report	Type: onabotulinumtoxinA Dilution: N/A Approach: IM Dose: Two per site (eight sites) Location: V1 and V2 branches	One idiopathic type 1 TN (left V2); after the failure of CBZ and then glycerol rhizotomy, burning pain spread in all three branches.	VAS Pain score significantly reduced from 82 to 54, 25, 25, and 45 at 7-day, 30-day, 60-day, and 90-day follow-up.	Mild paresis of the left frontal muscle.
[87] Türk et al., 2005	Open-label study	Type: onabotulinumtoxinA Dilution: 50 U/mL Approach: N/A Dose: 50 U per site (two sites) Location: one site above and one site below the zygomatic arch	Eight patients with idiopathic type 1 TN refractory to medical treatment.	Significant reduction in pain intensity and frequency at 1-week, 2-month, and 6-month follow-up. No quantitative data.	One patient reported chewing impairment for 3–4 days; one patient reported dysesthesia affecting the site treated.
[88] Piovesan et al., 2005	Open-label study	Type: onabotulinumtoxinA Dilution: N/A Approach: SC Dose: mean 3.22 U/cm^2^ Location: “follow the pain” technique	13 patients with TN (three performed previous surgery for TN). There was no information regarding the etiology and type of TN.	Significant reduction in pain and pain area in every branch studied. Maximum effect was observed at 20-day and 30-day follow-up. There was a positive correlation between pain area and intensity of pain. The effect lasted >60 days.	None
[89] Volcy et al., 2006	Case report	Type: N/A Dilution: N/A Approach: IM Dose: 7.5 U Location: in the left masseter and zygomatic muscles	One patient with idiopathic Type I TN (left V2)	Significant reduction in pain (over 90% reduction) over two months; the procedure was repeated two other times.	N/A
[90] Boscá-Blasco et al., 2006	Case series	Type: onabotulinumtoxinA Dilution: 25U/mL Approach: IM Dose: 15–17.5 U Location: orbicularis oculi	Four patients with painful convulsive tic (hemifacial spasm and classical TN); the type was not reported.	Significant reduction in pain (not quantified); 3–5 months duration.	None
[91] Zúñiga et al., 2008	Open-label study	Type: onabotulinumtoxinA Dilution: N/A Approach: SC Dose: 20–50U Location: in the trigger zone and painful areas.	12 patients with idiopathic TN. Did not specify the type of TN.	Pain significantly reduced from 8.83 ± 1.19 to 4.08 ± 4.44 after 8 weeks. The paroxysms reduced from 23.42 ± 13.5 to 8.67 ± 12.4 at the 8-week follow-up. Two patients did not respond to the treatment. Complete pain relief for 7 weeks and then gradual resumption of pain. Higher doses correlated with an earlier onset of action. Pain free for 60 days; at the 12-week follow-up pain gradually reprised.	Facial asymmetry in only one case
[92] Ngeow et al., 2010	Case report	Type: onabotulinumtoxinA Dilution: 40U/mL Approach: SC Dose: 40–60 U Location: in the right mental nerve territory and in the right nasal trigger zone.	One patient with idiopathic type 1 TN (right V2-V3); 14 neurectomies were performed and she later developed persistent neuropathic pain.	Complete resolution of pain for five months in the right nasal region; partial response in the right mental region. 5 months duration. Pain reduction is not quantified.	Facial asymmetry
[93] Bohluli et al., 2011	Open-label study	Type: N/A Dilution: N/A Approach: N/A Dose: 50–100 U Location: in the trigger zones.	15 patients with type 1 TN; not specified etiology.	Significant pain and frequency reduction at 1-week and 1-month follow-up. The duration of the effect was reported to be up to 6 months.	Facial asymmetry in three cases (one severe paralysis)
[94] Shehata et al., 2012	Randomized, double-blinded, placebo-control study	Type: onabotulinumtoxinA Dilution: 50 U/mL Approach: SC Dose: 40–60 U, 5 U per site Location: “follow the pain” technique	20 idiopathic TN patients; 10 treated with BoNT/A and 10 with placebo. Not specified the type.	Pain significantly reduced in the group treated with BoNT/A (6.5 NRS mean reduction) at the 12-week endpoint; the placebo group did not improve (0.3 NRS mean reduction). Paroxysm frequency significantly reduced at the two-week follow-up and persisted till the endpoint. No correlation between the dose and the pain reduction or paroxysm frequency.	The group treated with BoNT/A presented four cases of facial asymmetry, one case of hematoma, one case of itching, and one case of pain in the injection site. The placebo group did not present facial asymmetry, but there were two cases of hematoma, 1 case of pain, and one case of itching in the injection site.
[95] Wu et al., 2012	Randomized, double-blinded, placebo-controlled study	Type: lanbotulinumtoxinA Dilution: 50 U/mL Approach: intradermal or intraoral Dose: 75 U, 5 U per site Location: “follow the pain” technique	40 patients with classical TN; 21 treated with BoNT/A and 19 with placebo. Not specified the type.	68.18% of patients in the BoNT/A group had > 50% pain reduction at 12-week endpoint; 15% of the patients in the placebo group had > 50% pain reduction at 12-week follow-up. The frequency in the BoNT/A group significantly reduced at 1-week follow-up and persisted to the endpoint.	Five patients in the BoNT/A group experienced facial asymmetry; three with patients presented edema in the injection site (one of them was in the placebo group).
[96] Zúñiga et al., 2013	Randomized, double-blinded, placebo-controlled study	Type: onabotulinumtoxinA Dilution: 50U/mL Approach: SC Dose: 50U Location: “follow the pain” technique	36 TN patients; 20 treated with BoNT/A and 16 with placebo. Classical and idiopathic. Not specified the type.	Pain significantly reduced from 8.85 to 4.9 and 4.75 in the BoNT/A group at 2-month and 3-month follow-up, respectively; in the placebo group the pain significantly changed from 8.19 to 6.63 and 6.94 at two-month and 3-month follow-up respectively. The number of paroxysms in the BoNT/A group significantly reduced from 29.1 to 7.1 at 3-month follow-up. In the placebo group paroxysms reduced from 31.06 to 21.25 per day at 3-month follow-up, although this difference was not statistically significant. Duration of BoNT/A effect was reported to be at least 3 months.	None
[97] Li et al., 2014	Open-label study	Type: lanbotulinumtoxinA Dilution: 50U/mL Approach: SC or intraoral Dose: 20–170 U, 2.5–5 U per site Location: “follow the pain” technique	88 patients with classical TN; the sample was then divided into three groups based on BoNT/A dose (<50, 50–100, >100). Not specified the type.	92% had at least >50% pain reduction at one moth follow-up; 100% and 77% had >50% pain reduction at 2-month and 3-month follow-up, respectively. At 14-month follow-up, 38.6% had still at least >50% improvement and 22% were completely pain free. No significative difference between the different dose groups.	Local swelling in three patients; muscle weakness in 10 cases.
[98] Wang et al., 2014	Open-label study	Type: lanbotulinumtoxinA Dilution: N/A Approach: N/A Dose: N/A Location: N/A	16 patients with classical TN. Not specified the type.	Pain significantly reduced from 9.12 ± 0.65 to 2.8 ± 1.36, 2.2 ± 1.26, 1.3 ± 1.45, 1.3 ± 1.45, and 1.2 ± 2.52 at 1-week, 2-week, 1-month, 3-month, and 6-month follow-up, respectively.	NA
[99] Zhang et al., 2014	Randomized, double-blinded, placebo-controlled study	Type: lanbotulinumtoxinA Dilution: 100U/mL Approach: intradermal or intraoral Dose: 25 or 75 U Location: “follow the pain” technique	84 patients with classical TN; 27 treated with 25 U and 29 treated with 75 U. 28 patients were treated with placebo. Not specified the type.	The responders (>50% pain reduction), at 8-week follow-up, were 70.4%, 86.2%, and 32.1% for the 25 U group, 75 U group, and placebo, respectively. There was a significant pain reduction in both groups treated with BoNT/A but not for the placebo group. There was no difference in efficacy between the 25 U and 75 U groups.	Three patients treated with BoNT/A (two in the 25 U group and one in the 75 U group) presented with facial asymmetry. Transient edema in the injection site in two patients treated with 25 U of BoNT/A.
[100] Xu et al., 2015	Open-label study	Type: lanbotulinumtoxinA Dilution: 25 U/mL Approach: intradermal or intraoral Dose: 88 ± 30 U in the > 70 years old group, 72 ± 33 in the <60 years old group. Dose range 30–200 U, 2.5–5 U per site. Location: “follow the pain” technique	64 patients with idiopathic TN; sub-analysis of >70 years old and <60 years old. Not specified the type.	Pain significantly reduced from 7.7 ± 2.2 to 4.4 ± 2.9 at 1-month follow-up. No differences between the two age groups.	Seven patients with minor side effects.
[101] Xia et al., 2015	Open-label study	Type: lanbotulinumtoxinA Dilution: 50U/mL Approach: intradermal or subcutanoeus Dose: N/A Location: N/A	87 patients with classical TN, only one branch involved. Not specified the type.	Pain reduced significantly from 6.59 to 1.95 at 8-week follow-up.	Local swelling in two patients; facial weakness in seven patients.
[102] Lunde et al., 2016	Case report	Type: N/A Dilution: 100 U/mL Approach: SC and intradermal Dose: 75 U, 2.5–5 U per site Location: along the course of the three branches.	One patient with idiopathic type II secondary TN (all the three left branches affected).	Complete resolution of pain after 19 days. Gradual reprisal of pain at week 9.	N/A
[103] Zhang et al., 2016	Open-label, randomized trial	Type: lanbotulinumtoxinA Dilution: 50 U/mL Approach: intradermal or intraoral Dose: 70–100 U if single injection or 50–70 U each session in case of 2 injection (2 weeks apart). Location: “follow the pain” technique	81 patients with classic TN; 44 treated with a single injection and 37 treated with two injections 2 weeks apart. Not specified the type.	90.9% and 61.4% of the single injection group had pain reduction of at least > 50% at 2-month and 6 -month follow-up, respectively; 83.3 and 51.4% % of the double injection group had at least > 50% pain reduction at 2-month and 6-month follow-up respectively. Pain reduced significantly in the first group from 7.99 ± 1.60 to 1.59 ± 2.17 and 3.02 ± 3.29 at 3-month and 6-month follow-up respectively; the second group had similar results with pain reduction from 8.27 ± 1.69 to 2.36 ± 3.01 and 4.32 ± 3.61 at 3-month and 6-month follow-up, respectively. There were no statistical differences between the two groups.	14 patients with unspecified side effect (seven in both groups).
[104] Türk et al., 2017	Open-label study	Type: onabotulinumtoxinA Dilution: 50 U/mL Approach: near nerve Dose: 50U per branch Location: “follow the pain” technique	27 patients with classical TN; not specified the type.	Pain significantly reduced from 9.7 ± 0.6 to 2.4 ± 3.1 and 1.6 ± 2.4 at two-month and six-month follow-up, respectively. 74.1% and 88.9% had >50% pain reduction at 2-month and 6-month follow-up respectively. 44.4% were pain-free at 6-month follow-up. The frequency improved from 217 ± 331.5 paroxysms per day to 54.8 ± 196.2 and 55.15 ± 196.2 at 2-month and 6-month follow-up.	One patient with facial asymmetry; two patients with masseter weakness.
[105] Wu et al., 2018	Case report	Type: lanbotulinumtoxinA Dilution: 100 U/mL Approach: IM (masseter muscle) and intraoral Dose: 50 U Location: “follow the pain” technique	One patient with type 1 classical TN	No significant improvement with intraoral injection; significant pain reduction after intramuscular injection in the masseter muscle with complete remission of pain after 2 weeks.	N/A
[106] Liu et al., 2018	Open-label study	Type: lanbotulinumtoxinA Dilution: 25 U/mL Approach: intradermal or intraoral Dose: 30–200 U Location: “follow the pain” technique	43 patients with classical TN distributed in 14 >80 years and 29 < 60 years. Not specified the type.	Pain reduced significantly from 8.5 and 8.0 to 4.5 and 4.0 for the >80 years and <60 years groups respectively at 1-month follow-up.	Four patients with facial asymmetry (two in each group).
[107] Caldera et al., 2018	Open-label study	Type: N/A Dilution: N/A Approach: intradermal Dose: 15–50 U Location: “follow the pain” technique	22 with type 1 and type 2 idiopathic TN patients with insufficient control with first-line treatment.	Pain significantly reduced from 7.41 ± 2.2 to 5.59 ± 2.7, 5.68 ± 2.6, 5.27 ± 3.2, 4.77 ± 3.7, and 5.32 ± 4.0 at 10-day, 20-day, 30-day, 60-day, and 90-day follow-up, respectively. No differences between high dose vs. low dose of BoNT/A.	None
[108] Crespi et al., 2019	Open-label study	Type: onabotulinumtoxinA Dilution: 50 U/mL Approach: into the sphenopalatine ganglia Dose: 25 U Location: into the sphenopalatine ganglia	10 patients with refractory classical Type 2 TN.	Pain significantly reduced from 6.0 (3.0–8.5) to 3.0 (0.0–9.0) at 5/8-week follow-up. Frequency of paroxysms did not reduce significantly. Concomitant persistent pain not improved after adjustment for multiplicity.	There were four cases of swelling, five cases of jaw dysfunction, two cases of facial asymmetry, one case of dry eye, one case of diplopia, one case of cutaneous rash, and one case of dysphagia.
[109] Wu et al., 2019	Retrospective	Type: lanbotulinumtoxinA Dilution: 50 U/mL Approach: intradermal or intraoral Dose: 2.5–5 U per site Location: “follow the pain” technique	104 patients with refractory classical TN. Not specified the type.	87 patients reported pain reduction > 50% at 8-day follow-up (41 had complete pain control); 17 patients reported mild improvement, no improvement, or worsening. Patients with age > 50 years had better outcomes.	17 patients reported facial asymmetry.
[110] Calejo et al., 2019	Case report	Type: N/A Dilution: N/A Approach: N/A Dose: 45U, 5 U per site and three sites per branch Location: “follow the pain” technique	One patient with secondary (multiple sclerosis) type 1 TN, with all the branches involved.	There was 75% pain reduction that lasted 3 months.	Facial asymmetry
[111] Yoshida et al., 2019	Open-label study	Type: onabotulinumtoxinA Dilution: 50 U/mL Approach: into the sphenopalatine ganglia Dose: 50 U Location: into the sphenopalatine ganglia	10 patients with classical TN (V2), refractory to submucosal BoNT/A injections. Not specified the type.	Pain significantly reduced from 8.1 ± 1.0 to 1.9 ± 1.4 at 4-week, 8-week, and 12-week follow-up. Pain frequency decreased from 19.4 ± 8.8 to 4.8 ± 5.4, 3.8 ± 5.5, and 4.8 ± 5.4 at 4-week, 8-week, and 12-week follow-up.	None
[112] Zhang et al., 2019	Retrospective	Type: lanbotulinumtoxinA Dilution: 50 U/mL Approach: intradermal or intraoral Dose: 1.25–5 U per site Location: “follow the pain” technique	152 patients with classical TN; subdivided in low dose (<40 U), medium dose (40–70 U), and high dose (>70 U). Not specified the type.	89.4% reported reduction in pain intensity of at least >50% at 2-week follow-up; the effect was sustained throughout the first 6 months of follow-up. Patients who received high and medium dose had cases of more complete pain relief than the low dose group (21.1% and 22.7% vs. 11.2%); however, the difference was not statistically significant, and the overall efficacy was similar. High dose was associated with longer duration effect of BoNT/A on pain. 58 patients had follow-up longer than 7 months without increment in pain scores.	21 patients presented facial asymmetry.
[113] Dinan et al., 2020	Case report study	Type: onabotulinumtoxinA Dilution: N/A Approach: intraoral and SC Dose: 30 U subcutaneously and 20 U intraorally Location: “follow the pain” technique	One patient with idiopathic type 1 TN (V2 and V3 involvement).	Complete pain relief 3 days after the treatment; 3-month duration.	Facial asymmetry
[114] Mingazova et al., 2021	Open-label study	Type: onabotulinumtoxinA Dilution: N/A Approach: SC and IM (masseter muscle) Dose: mean dose 80 U (35 frontal; 20 U temporal region; 10–16 in middle and lower part of the face, 10 U into the masseter muscle) Location: along the course of the branches.	20 patients with classical and idiopathic TN. Not specified the type.	Pain intensity did not reduced significantly at 1-month follow-up (8.5 vs. 7.2) while at 2-month and 3-month follow-up this difference was significant (6.1 and 4.9, respectively). The frequency of the paroxysms significantly reduced from 31.2 per day to 22.5, 17.7, and 9.2 at 1-month, 2-month, and 3-month follow-up, respectively. 50% and 38% of the patients were pain-free at 90-days and 115-days follow-up.	N/A
[115] Yoshida et al., 2021	Open-label study	Type: onabotulinumtoxinA Dilution: 50 U/mL Approach: SC and intraoral Dose: 43.1 ± 5.3 U Location: “follow the pain” technique	28 patients with TN. Not specified etiology and type.	Pain significantly reduced from 89.3 ± 7.5 to 35.1 ± 6.6, 25.9 ± 6.8, 20.8 ± 7.0, and 19.5 ± 7.3 at 2-week, 4-week, 8-week, and 12-week follow-up. Frequency significantly reduced from 19.1 ± 7.7 paroxysms per day to 9.8 ± 4.9, 5.6 ± 3.5, 4.2 ± 2.9, and 3.7 ± 2.6 at 2-week, 4-week, 8-week, and 12-week follow-up	Muscle weakness and and tenderness at the injection site; not quantified.
[116] Asan et al., 2022	Retrospective	Type: onabotulinumtoxinA Dilution: N/A Approach: SC and intraoral Dose: 32.5–50 U, 2.5 U per site Location: “follow the pain” technique	53 patients with idiopathic TN (22) or secondary TN (31) due to MS (RR or PP); Type 2 TN present in 12 patients in the first group and 17 patients in the second group.	Pain reduced significantly from 9 to 6. The idiopathic TN group had significant pain reduction from 8 to 7; the MS TN group had significant pain reduction from 9 to 5. Efficacy was higher in the type 2 TN (62% vs. 33%; *p* 0.047).	BoNT/A was administered also in the contralateral side to prevent facial asymmetry. Only two patients reported facial asymmetry.
[117] Pearl et al., 2022	Case report	Type: N/A Dilution: 50 U/mL Approach: intraoral Dose: 20 U Location: in the left mental foramen	Two patients with type 1 TN (one classical left V3 TN; one secondary left V3 TN due to MS).	Patient 1 had complete pain relief for 6 weeks, 10 weeks, 5 months and 18 months after the first, second, third, and fourth treatments, respectively. Patient 2 had complete pain relief for 5 months and 18 months after the first and second treatments, respectively.	N/A
[118] Xiromerisiou et al., 2023	Retrospective	Type: onabotulinumtoxinA Dilution: 50–25 U/mL Approach: SC Dose: 5–2.5 U per site Location: “follow-the pain” approach	15 patients with TN (11 with idiopathic or classical TN and four with secondary TN). Not specified the type.	Pain decreased from a baseline VAS of 9.3 ± 1.1 to 3.7 ± 1.2 at 2-week follow-up.	Five patients with mild and transient erythema and/or tenderness, resolved within 48 h. No facial asymmetry.
[119] Tereshko et al., 2023	Open-label study	Type: onabotulinumtoxinA Dilution: 100 U/mL Approach: SC and intradermal Dose: 29.7 ± 11.4 U Location: “follow-the pain” technique, along the course of the branches involved.	40 TN patients (18 type 1 TN and 22 type 2 TN); 18 patients with classical TN and 22 with idiopathic TN.	Pain reduced from 8.1 ± 1.4 (VAS) to 3.3 ± 2.3 and 3.4 ± 2.4 at the 1-month and 3-month follow-ups (*p* < 0.001). Type 1 TN and type 2 TN groups had baseline pain scores of 7.8 ± 1.65 and 8.4 ± 1.1, respectively. Pain significantly improved (*p* < 0.001) in both groups to 3.1 ± 2.3 (type 1 TN) and 3.5 ± 2.3 (type 2 TN) at the 1-month follow-up and to 3.2 ± 2.5 (type 1 TN) and 3.6 ± 2.5 (type 2 TN) at the 3-month follow-up. There was no difference between the two groups (*p* 0.345).	14 patients had mild transient facial asymmetry (six type 1 TN and eight type 2 TN patients).

## Data Availability

No new data were created or analyzed in this study. Data sharing is not applicable to this article.

## Abbreviations

AH	hemagglutinin proteins
BoNT	botulinum neurotoxin
CBZ	carbamazepine
CGRP	calcitonin gene-related peptide
COX	cyclooxygenase
DRG	dorsal root ganglia
EMA	European Medicine Agency
ERK	extracellular signal-regulated kinase
FDA	Food and Drug Administration
GABA	gamma aminobutyric acid
HC	heavy chain
IL	interleukin
KD	kilodalton
lanA	lanbotulinumtoxinA
LC	light chain
NAH	non hemagglutinin proteins
NAPs	non-toxic neurotoxin-associated proteins
NA	not available
NOS2	nitric oxide synthetase 2
onaA	onabotulinumtoxinA
PAG	peri-aqueductal gray
SNAP25	synaptosome-associated protein of 25 KDa
SNARE	SNAP receptors
SUNHA	short-lasting unilateral neuralgiform headache attacks
TLR2	toll-like receptor 2
TNF	tumor necrosis factor
TN	trigeminal neuralgia
TRPV1	transient receptor potential cation channel subfamily V member 1
VAMP2	vesicle-associated membrane protein 2
